# Ellagic Acid Activated PPAR Signaling Pathway to Protect Ileums Against Castor Oil-Induced Diarrhea in Mice: Application of Transcriptome Analysis in Drug Screening

**DOI:** 10.3389/fphar.2019.01681

**Published:** 2020-01-31

**Authors:** Jianqing Chen, Hongliang Yang, Zunlai Sheng

**Affiliations:** ^1^ College of Veterinary Medicine, Northeast Agricultural University, Harbin, China; ^2^ College of Animal Science and Technology, Northeast Agricultural University, Harbin, China; ^3^ Heilongjiang Key Laboratory for Animal Disease Control and Pharmaceutical Development, Northeast Agricultural University, Harbin, China

**Keywords:** ellagic acid, transcriptome, PPAR signaling pathway, castor oil, diarrhea

## Abstract

**Background:**

Acute diarrhea is still a common and serious disease. The causes of acute diarrhea are very complicated. Therefore, we need to find a medicine to control diarrhea symptoms, save time for diagnosis of pathogens, and prevent drug abuse. Ellagic acid (EA), a natural polyphenol drug, has anti-diarrhea effects. However, the action mechanisms of EA for non-specific diarrhea have not been characterized.

**Materials and Methods:**

To study the mechanisms of EA, mice were divided into four groups. Group C were intraperitoneally injected with 0.1 ml physiological saline and orally given 0.2 ml physiological saline, and then after experiment began 0.5 h, orally administered 0.3 ml physiological saline. Group D were intraperitoneally injected with 0.1 ml physiological saline and orally given 0.2 ml castor oil, and then after experiment began 0.5 h, orally administered 0.3 ml physiological saline. Group E were intraperitoneally injected with 0.1 ml physiological saline and orally given 0.2 ml castor oil, and then after experiment began 0.5 h, orally administered 0.3 ml EA (10 mg/ml). Group V were intraperitoneally injected with 0.1ml GW9662 (1m g/ml) and orally given 0.2 ml castor oil, and then after experiment began 0.5 h, orally administered 0.3 ml EA (10 mg/ml). Transcriptome were performed on ileum tissues of mice in group D and E. Histological examination and qRT-PCR were performed on ileum tissues of mice in group C, D, E, and V.

**Results:**

We found that a total of 273 differentially expressed genes (DEGs) were obtained, including 160 up-regulated DEGs and 113 down-regulated DEGs. The DEGs were enriched in 458 Gene Ontology (GO) terms and 15 Kyoto Encyclopedia of Genes and Genomes (KEGG) pathways, respectively. The peroxisome proliferator activated receptor (PPAR) signaling pathway was the most significantly enriched in KEGG pathways. We used the PPAR-specific antagonist GW9662 to validate the anti-diarrhea and anti-inflammatory effect of EA in group V compared with group E. Conclusively, EA protected ileums against castor oil-induced inflammation and diarrhea by activating the PPAR signaling pathway and a method was used to study the mechanism of EA.

## Introduction

Diarrhea is the passage of liquid or loose stools more frequently than normal for an individual (Vrese and Offick, 2010). Acute diarrhea is a common and serious disease worldwide. Miller et al. (2002) reported that nosocomial clostridium difficile-associated diarrhea is a nosocomial infectious complication, has relationship with substantial morbidity and mortality, and causes a financial burden on healthcare institutions in Canada (Miller et al., 2002). In USA, diarrhea is an important cause of morbidity in this insured population of young children (Zimmerman et al., 2001). Curcio reviewed resent studies and found that there is a high incidence of clostridium difficile-associated diarrhea in developing countries (Africa–Middle East, developing Asia, Latin America, and China) (Curcio et al., 2019).

The causes of acute diarrhea are very complicated. Toxicosis, endocrine dyscrasia, viruses, bacteria, parasite, and medications can cause acute diarrhea. Acute infectious diarrhea is one of the most common diseases in pediatric age with relevant burden both in high- and in low-income countries (Lo Vecchio et al., 2019). Studies have reported that acute diarrhea was accompanied by an increase in inflammatory responses (Mazzolin et al., 2013; Dong et al., 2019) and oxidative stress (Jabri et al., 2016a). More importantly, castor oil-induced intestinal hypersecretion had a physiological response similar to acute diarrhea in the intestine (Mazzolin et al., 2013; Jabri et al., 2016a; Jabri et al., 2016b). Models used in studying diarrhea often employ castor oil in mice (Robert and Rao, 1996) or rats (Amabeoku and Bamuamba, 2010). Symptoms of castor oil-induced diarrhea are non-specific and these models have been widely used in the screening of natural anti-diarrheal drugs.

To treat acute diarrhea, the first thing is to find the causes. Therefore, screening non-specific anti-diarrhea drugs from natural plants will save time for finding diarrhea causes and targeted medicines. For example, Pandey et al. (2017) reported that 50% ethanolic extracts of grilled and dried fruits effectively inhibited diarrhea (Pandey et al., 2017). Due to increasing interest in plant-based traditional medicines, many researches are interested in exploring their activities and mechanisms of action (Zhao et al., 2018). Natural products boast many advantages, such as low cost, ease of availability, the ability to circumvent drug resistance, and relief of diarrhea symptoms. These advantages enable earlier pathogen diagnosis and treatment. For instance, many natural antidiarrheal plants contained ellagic acid (EA) (Singh et al., 2017). EA is the dilactone of hexahydroxydiphenic acid and a natural phenol antioxidant found in fruits and vegetables. EA has potential anti-inflammatory and antioxidant properties (Priyadarsini et al., 2002; Yim et al., 2016; Verotta et al., 2018) and has been shown to have a preventive effect against many diseases, such as neurodegenerative diseases, cancer and diarrhea *in vitro* and *in vivo* (Buniatian, 2003; Kannan and Quine, 2012; Ahmed et al., 2016).

Peroxisome proliferator-activated receptors (PPAR) belong to the nuclear receptor superfamily of ligand-inducible transcription factors. PPARs are classified into three subtypes α, β/δ, and γ. Studies showed that the biological functions of PPARs are complex. Yaribeygi et al. (2018) reported that activated PPAR-α restored anti-oxidant defense systems and improved diabetes-induced oxidative stress (Yaribeygi et al., 2018). Intriguingly, Zeinali et al. (2017) suggested that chrysin (CH), a plant polyphenolic compound, which acts as an agonist of PPAR-γ, can be used as an anti-inflammatory and anti-oxidative agent in immunopathological and physicochemical injuries (Zeinali et al., 2017). Cadmium caused high levels of inducible nitric oxide synthase (iNOS) activity, nitric oxide (NO) content, and apoptosis *via* PPAR-γ/PI3K/Akt pathway in chicken pancreas (Jin. et al., 2018). Compared to dinitrobenzene sulfonic acid (DNBS)-treated PPAR-α wild-type (WT) mice, DNBS-treated PPAR-α knockout mice (PPAR-αKO) mice experienced more hemorrhagic diarrhea, more weight loss, higher rate of the extent and severity of the histological signs of colon injury (Cuzzocrea et al., 2004). Sarnelli et al. (2018) also found that palmytoilethanolamide, *via* PPAR-α-dependent mechanism, resulted in a significant antidiarrheal activity in WT rats (Sarnelli et al., 2018). However, the effect of EA on the PPAR signaling pathway remains unclear.

Transcriptome analysis is a recently developed deep sequencing technique and involves an informatics approach to solve an experimental limitation (Martin and Wang, 2011). Transcriptome has been developed for many research fields, such as toxicology (Chen et al., 2018; Chen et al., 2019); Li et al. (2018) and Skaria et al. (2019) reported that transcriptome was used to investigated molecular mechanism of drugs (Li et al., 2018; Skaria et al., 2019). The purpose of this study was to evaluate the protective mechanism of EA against diarrhea by transcriptome analysis in mouse models. Furthermore, histopathological examination and redox biomarkers were determined to study the anti-inflammatory and antioxidative effects of EA. We used the PPAR-specific antagonist GW9662 to validate the anti-inflammatory effects of EA. Transcriptome and qRT-PCR results showed that the PPAR signaling pathway was involved in the preventive mechanism of EA against castor oil-induced diarrhea.

## Materials and Methods

### Animals and Treatments

BALB/c mice were purchased from Harbin Medical University (Harbin, China) and raised in the experimental animal facility of Northeast Agricultural University. All experimental processes about animals complied with EU Directive (2010/63/EU) and were approved by the Ethics Committee of Northeast Agricultural University of China (Protocol number: SRM-06). After being acclimated for two weeks, twenty-one days old healthy mice (20.1 ± 0.5 g) were randomly divided into four groups (ten mice each group). The control group (group C) were intraperitoneally injected with 0.1ml physiological saline and orally given 0.2 ml physiological saline, and then after experiment began 0.5 h, orally administered 0.3 ml physiological saline. The diarrhea group (group D) were intraperitoneally injected with 0.1ml physiological saline and orally given 0.2 ml castor oil, and then after experiment began 0.5 h, orally administered 0.3 ml physiological saline. The EA group (group E) were intraperitoneally injected with 0.1ml physiological saline and orally given 0.2 ml castor oil, and then after experiment began 0.5 h, orally administered 0.3 ml EA (10 mg/ml). The verification group (group V) were intraperitoneally injected with 0.1ml GW9662 (1 mg/ml) and orally given 0.2 ml castor oil, and then after experiment began 0.5 h, orally administered 0.3 ml EA (10 mg/ml).

Castor oil can cause frequent stooling within 4 h. Each group mice were placed in a beaker (5000 ml) with a weighed filter paper at the bottom for observation. Filter paper in each beaker was also changed at the same time and weighed to obtain the stool mass. The doses and pretreatment times were obtained from preliminary studies in our laboratory. All experiments were carried out once a day for 3 consecutive days in a quiet laboratory and the ambient temperature was 20.5 ± 1°C. At the third day, we sacrificed the mice for experiments. Ileum samples were collected from each group and washed with physiological saline solution (0.9% NaCl) on ice-cold plates. Samples from group D and E were prepared for mRNA sequencing. All the mouse ileums of four groups were collected for morphological examination, oxidative stress biomarkers and proinflammatory factor kit and qRT-PCR.

### mRNA Sequencing and Analysis

Total RNA was extracted with TruSeq Stranded Total RNA Library Prep Kit (Illumina, USA). RNA concentration and total quantity were detected by Invitrogen Qubit 3.0 Spectrophotometer (Thermo Fisher Scientific, USA); RNA purity was checked using the Nanodrop 2000 (Thermo Fisher Scientific, USA); and RNA integrity was checked by Agilent 2100 Bioanalyzer (Agilent Technologies, USA). mRNA was obtained through purifying total RNA, and was broken into fragments of 100–300 bp. Reverse Transcribe was performed to synthesize First Strand cDNA using SuperScript IV Reverse Transcriptase (Thermo Fisher Scientific, USA). Second strand cDNA synthesis was subsequently performed using DNA Polymerase I and RNase H. Then addition of adenlylate 3′ ends, connection sequencing adapters, selection library size, PCR amplification and checking library quality were performed in turn. Sequencing was performed on the Illumina Hiseq 2500 (Illumina, USA) and raw data was obtained. Raw data were first processed through in-house Perl scripts. Clean data were obtained by removing reads containing adapter sequences, poly-N sequences, and low-quality reads from raw data. The Q20, Q30, and GC content of the clean data were also calculated.

Reference genome and gene model annotation files were downloaded from the GenBank directly. An index of the reference genome was built using Bowtie v2.2.3. Paired-end clean reads were aligned using TopHat v2.0.12. Differential expression analysis of the two groups was performed using the DESeq R package (1.18.0). DESeq provided statistical routines for determining differential expression in digital gene expression data using a model based on the negative binomial distribution. Resulting P-values were adjusted using the Benjamini and Hochberg’s approach for controlling the false discovery rate. Genes with an adjusted *P*-value < 0.05 found by DESeq were assigned as differentially expressed. GO and KEGG enrichment analysis of differentially expressed genes (DEGs). Gene Ontology (GO) enrichment analysis of differentially expressed genes (DEGs) was implemented by the GOseq R package, in which gene length bias was corrected. GO terms with corrected *P*-value less than 0.05 were considered significantly enriched by DEGs. KEGG was a database for understanding high-level functions and utilities of the biological system, such as the cell, the organism and the ecosystem, from molecular-level information, especially large-scale molecular datasets generated by genome sequencing and other high-throughput experimental technologies (http://www.genome.jp/kegg/). We used KOBAS software to test the statistical enrichment of DEGs in KEGG pathways.

### Morphological Examination in the Ileum

Ileum samples were cut into 0.5 cm × 0.5 cm tissue blocks, fixed in 10% formaldehyde, and embedded in paraffin. Sections were then stained with hematoxylin and eosin. Tissue slices were observed under a microscope by a pathologist blinded to the experiment (Hu et al., 2018).

### Redox Biomarker Determinations

Ileum samples were homogenized in physiological saline, centrifuged at 3,000 g for 15 min, and supernatants were collected. The activities of superoxide dismutase (SOD) and glutathione peroxidase (GPx) and the content of malondialdehyde (MDA) were determined. Commercial assay kits for SOD (Superoxide Dismutase assay kit (A001-1)), GPx (Glutathione Peroxidase (GPX) assay kit (A005)) and MDA (Malondialdehyde (MDA) assay kit (A003-1)) were provided by Jiancheng Biotechnology Research Institute (Nanjing, China) (Su et al., 2018).

### qRT-PCR Analysis

To validate the reliability of the RNASeq results, we selected Ccr6, Cd36, Cyp2e1, GPx, H2-Ob, interleukin (IL)-1β, IL-6, NF-κB, PPAR-γ, Sod (SOD), and TNFα genes for qRT-PCR. A housekeeping gene (β-actin) was used as a reference. Primer information for qRT-PCR are shown in **Table 1**. Reactions were incubated in a Light Cycler^®^ 480 System (Roche, Basel, Switzerland). Reactions contained 10 μl 2×SYBR Green I PCR Master Mix (Roche, Basel, Switzerland), 2 μl of diluted cDNA, 0.4 μl of each primer (10 μM), 0.4 μl 50×ROX reference Dye II and 6.8 μl PCR-grade water. PCR cycling conditions were: one cycle at 95 °C for 30 s, followed by 40 cycles at 95 °C for 15 s and 60 °C for 30 s. The amplification efficiency for each gene was determined using the DART-PCR program. Relative mRNA abundance was calculated according to the Pfaffl method (Pfaffl, 2001).

**Table 1:** Gene-special primers used in qRT-PCR.

Gene	Forward (5 → 3)	Reverse (5 → 3)
Ccr6	GTGTGGCAGTGTGGTTCATCTCC	GTGGCTCACAGACATCACGATCC
Cd36	GCGACATGATTAATGGCACAGACG	CCGAACACAGCGTAGATAGACCTG
Cyp2e1	AAGGACGTGCGGAGGTTTTCC	TACATGGGTTCTTGGCTGTGT
GPx	CGCTTTCGTACCATCGACATC	GGGCCGCCTTAGGAGTTG
H2-Ob	CACAACCTGCTGCTCTGCTCTG	GACCTCTCCTCCTGTCCATTCCG
IL-1β	GCAACTGTTCCTGAACTCAACT	ATCTTTTGGGGTCCGTCAACT
IL-6	GGAGCCCACCAAGAACGATA	ACCAGCATCAGTCCCAAGAA
NF-κB	TCTCTATGACCTGGACGACTCTT	GCTCATACGGTTTCCCATTTAGT
PPAR-γ	CCAGAGCATGGTGCCTTCGCT	CAGCAACCATTGGGTCAGCTC
Sod	GTG ATTGGG ATTGCGCAG TA	TGGTTTGAG GGTAGCAGATGAGT
TNF-α	CCCTCACACTCAGATCATCTTCT	GCTACGACGTGGGCTACAG
β-actin	GTGCTATGTTGCTCTAGACTTCG	ATGCCACAGGATTCCATACC

## Statistical Analysis

Statistical analysis was performed using SPSS software (version 20.0, SPSS Inc., Chicago, IL, USA). Statistical significance was evaluated by one-way analysis of variance (ANOVA) followed by Tukey’s multiple comparison test. Data are expressed as mean ± standard deviation (SD).

## Results

### Animal Observations

We used the indexes of mass of stool, fecal output, onset of diarrhea, number of animals exhibiting diarrhea, and percentage episode inhibition to evaluate diarrhea symptoms, as shown in **Table 2**. No diarrhea symptoms were observed in mice of group C. Mass of stool were significantly decreased in group E (*P* < 0.01) and V (*P* < 0.05) compared with that in group D. EA effectively inhibits diarrhea onset of diarrhea in group E compared with that in group D (*P* < 0.01), while there was no difference in Onset of diarrhea between group D and group V (*P* > 0.05).

**Table 2:** Effect of EA on castor oil-induced diarrhea in mice.

Group	C	D	E	V
Mass of stool (Mean ± SD (g))	0	0.75 ± 0.12	0.23 ± 0.02**	0.65 ± 0.03*
Fecal output (%)	0	100	30.67	86.67
Onset of diarrhea (Mean ± SD (min))	–	30.12 ± 1.96	84.74 ± 3.84**	35.33 ± 6.20
No. of animals exhibiting diarrhea	0	10/10	2/10	10/10
Percentage Episode inhibition (%)	–	0	80.0	0

*represented significant difference (P < 0.05). **represented significant difference (P < 0.01). Statistical significance was evaluated by one-way analysis of variance (ANOVA) followed by Tukey’s multiple comparison test.

### Morphological Structure

**Figure 1:**
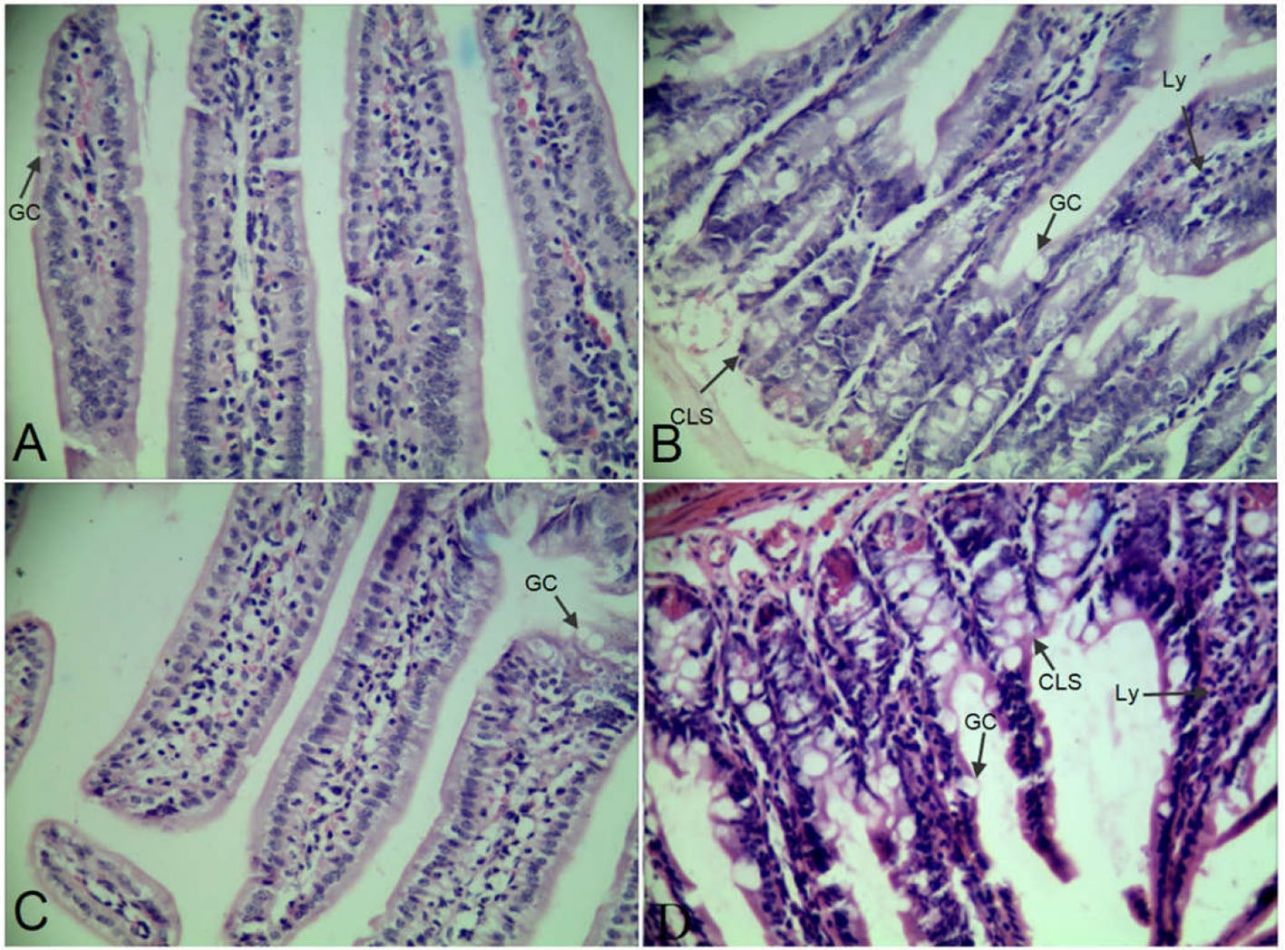
Ileums slices using HE staining. **(A**–**D)** were in the 400 × magnification.

Morphological structure of group C, D, E, and V were shown in **Figure 1**. The epithelial cell structures of ileums were arranged neatly and clear in group C (**Figure 1A**) and E (**Figure 1C**) compared with group D (**Figure 1B**) and V (**Figure 1D**). The numbers of goblet cells (GC) were increased and lots of lymphocytes (Ly) were aggregated in group D (**Figure 1B**) and V (**Figure 1D**). The tissues showed cellulose-like swelling (CLS), uneven staining, and inflammatory exudation in group D (**Figure 1B**) and V (**Figure 1D**).

### Redox Biomarker Determination

**Figure 2:**
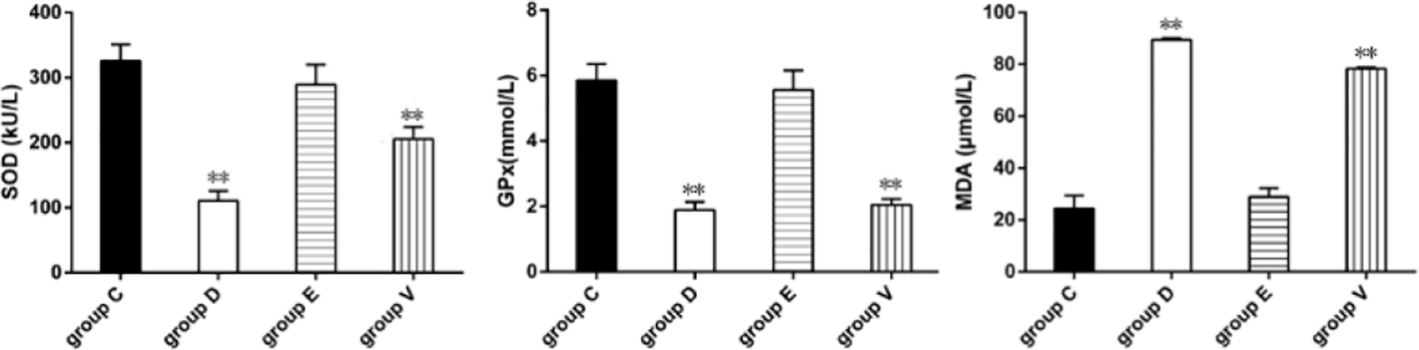
Determination of redox biomarker. **represented significant difference (*P* < 0.01). Statistical significance was evaluated by one-way analysis of variance (ANOVA) followed by Tukey’s multiple comparison test.

We evaluated the systemic oxidative balance by redox biomarker determination in groups C, D, E and V. Levels of SOD, GPx, and MDA in ileum tissues of group C, D, E, and V mice were shown in **Figure 2**. Castor oil induced oxidative damage of the ileums, while EA significantly alleviated castor oil-induced oxidative damage. SOD activity was significantly reduced in group D and V (*P* < 0.01). There were no differences between groups C and E. Compared to group C, castor oil significantly decreased GPx content in group D and V (*P* < 0.01), with no significant difference in group E (*P* > 0.05). Compared to group C, castor oil significantly increased MDA content in group D and V (*P* < 0.01).

### Proinflammatory Factor Determination

**Figure 3:**
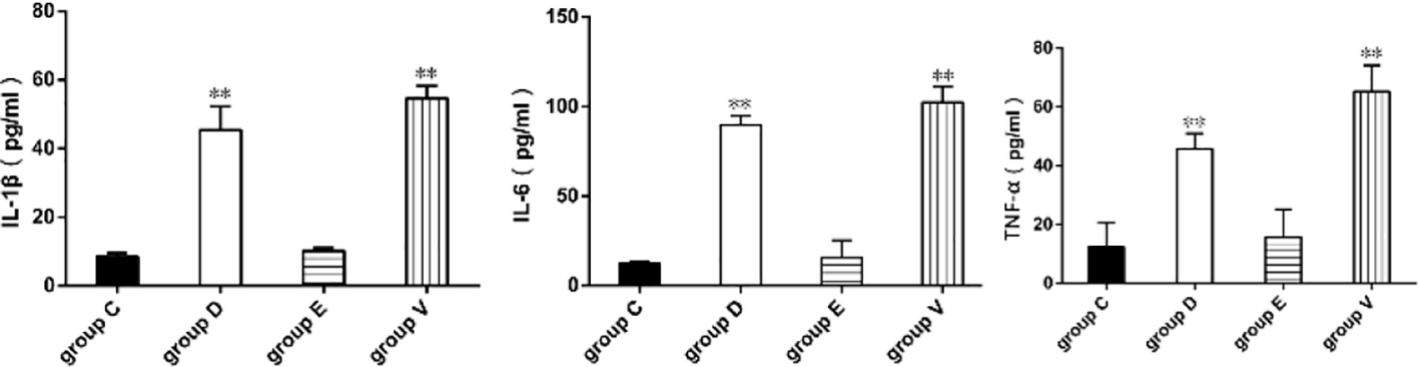
Determination of proinflammatory factors. **represented significant difference (*P* < 0.01). Statistical significance was evaluated by one-way analysis of variance (ANOVA) followed by Tukey’s multiple comparison test.

IL-1β, IL-6, and TNF-α followed similar trends in the four experimental groups, as shown in **Figure 3**. IL-1β, IL-6, and TNF-α levels were higher in group V and D compared those with group C (*P* < 0.01), while IL-1β, IL-6 and TNF-α levels were no significantly increased in group E compared with that in group C (*P* > 0.05).

### mRNA Sequencing and Analysis

Libraries from the group D and E were mapped using TopHat2. The characteristics of these libraries are summarized in **Table 3**. There were 106,567,752 and 100,936,974 raw reads and 116,141,362 and 104,686,394 raw reads were obtained from group D and E respectively. A total of 106,530,110 and 100,899,108 clean reads were obtained from group D and 116,125,974 and 104,665,316 clean reads were obtained from the group E. Clean base ratios were 99.96% in group D and 99.99 and 99.98% in group E. Parameters of Q20 were 97.51 and 97.12% in group D and 97.69 and 97.57% in group E and Q30 were 93.68, and 92.74% in group D and 94.21 and 93.95% in group E.

**Table 3:** Summary statistics of the transcriptome sequencing from group D and group E.

Sample	Raw reads	Clean reads	Clean base ratio (%)	Error rate(%)	Q20 (%)	Q30 (%)	GC content(%)
**D1**	106,567,752	106,530,110	99.96	0.0205	97.51	93.68	53.58
**D2**	100,936,974	100,899,108	99.96	0.0356	97.12	92.74	53.71
**E1**	116,141,362	116,125,974	99.99	0.0286	97.69	94.21	52.29
**E2**	104,686,394	104,665,316	99.98	0.0217	97.57	93.95	52.44

### Differential Expression Analysis

**Figure 4:**
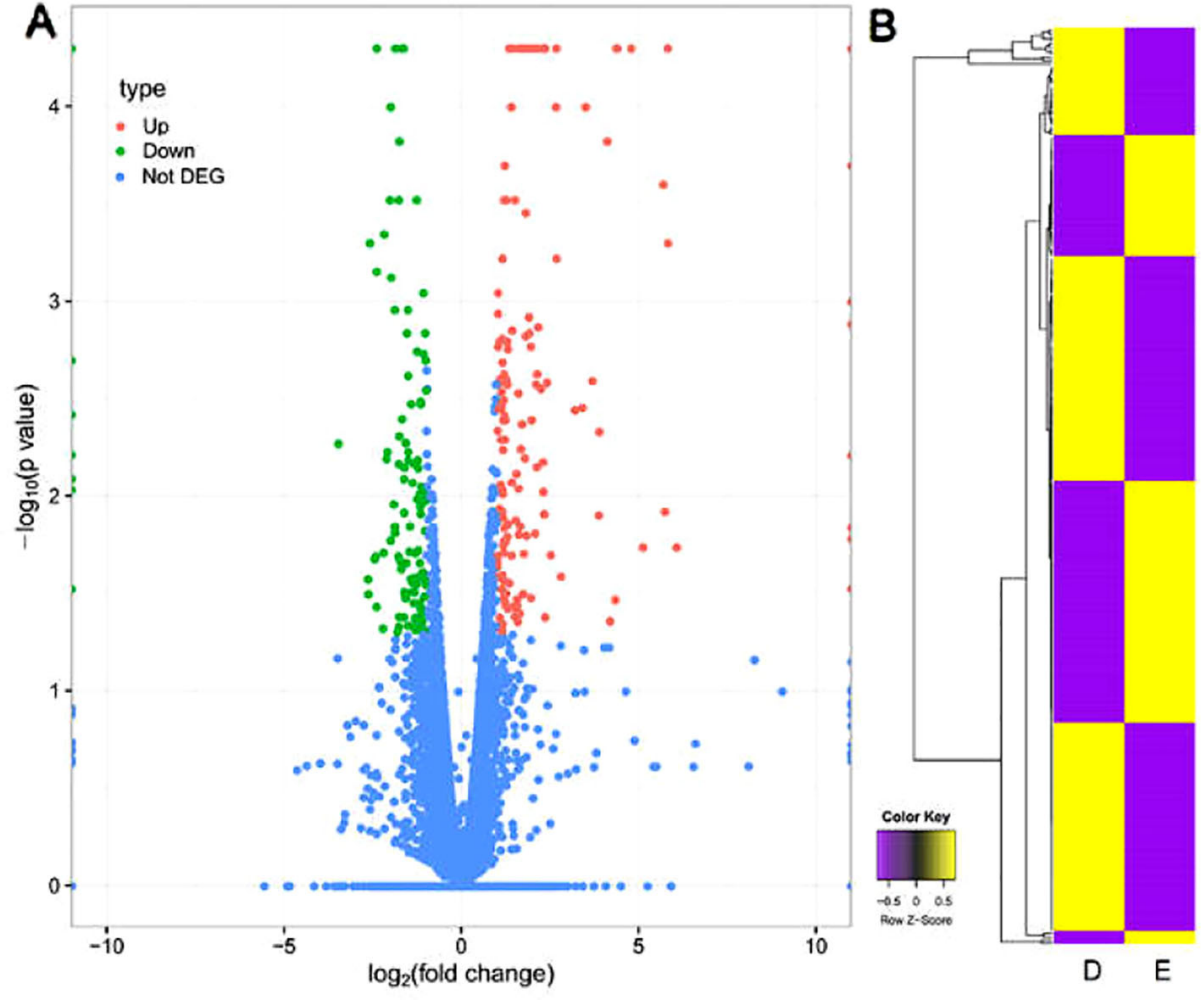
The figure of DEGs. **(A)** Volcano plot of distribution trends for DEGs in group D and group E. The log_2_ [fold change (group D/group E)] indicated the mean expression level for each gene. Each dot represented one gene. Red dots represented upregulated genes and green dots represented down-regulated genes. Blue dots represented genes with no differential expression. **(B)** Hierarchal clustering heat map of group D and group E.

RNA-Seq reads mapped to mouse reference genome were aligned and their relative abundances were estimated using TopHat2. According to the statistical analysis of unigene data with differentially expressed genes sequencing, genes significantly and differentially expressed between group E and group D were identified. A fold-change in gene expression >2 and *P* < 0.05 were considered to be differential expressed genes (DEGs). The results revealed that 273 genes were differentially expressed, including 160 up-regulated and 113 down-regulated genes. The volcano plot in **Figure 4A** showed the distribution trends for DEGs (green spots represented down-regulated genes; red spots represented up-regulated genes) and non-DEGs (blue spots).

DEGs were analyzed by hierarchal clustering heatmap analysis, and genes with the same or similar expression behavior were clustered (**Figure 4B**). The hierarchal clustering heatmap were shown log_2_(transformed FPKM values) for all 273 DEGs in group D and group E. Each column represented group D or E and each row represented a DEG (yellow denoted upregulation; purple denoted downregulation; and the color scale was at the bottom). Expression values are mean-centered.

### GO and KEGG Enrichment Analysis of DEGs

**Figure 5:**
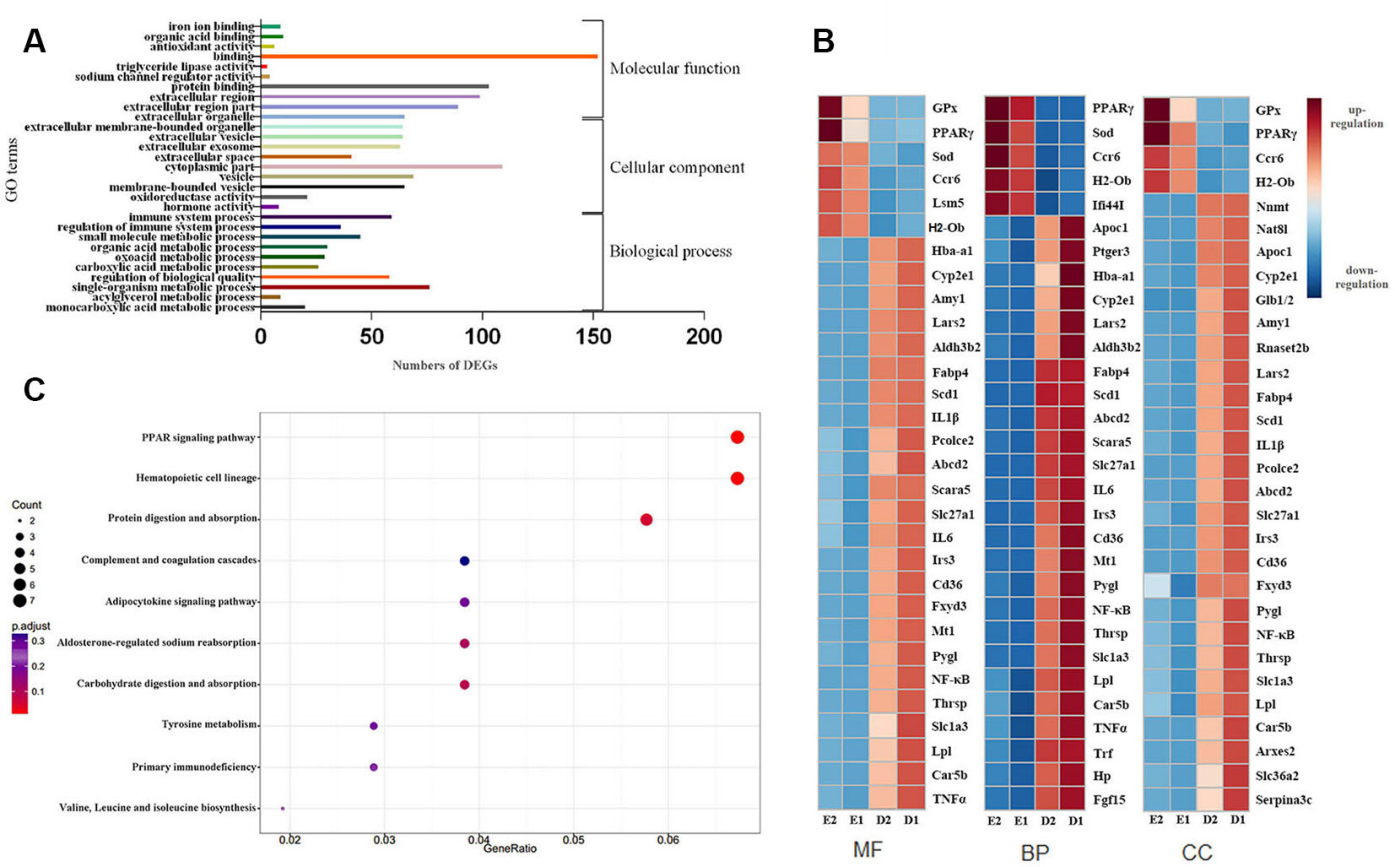
GO and KEGG analysis of DEGs. **(A)** The top 10 GO terms of BP, MF, and CC. **(B)** The top 30 DEGs heatmaps of BP, MF, and CC. **(C)** 15 pathways of DEGs using KEGG enrichment analysis.

A total of 458 GO terms between groups D and E were significantly enriched with GO analysis, including 358 biological process (BP) terms, 51 cellular component (CC) terms, and 49 molecular function (MF) terms. We selected the top 10 GO terms from each of the BP, CC and MF subgroups based on significance (*P*-value), as shown in **Figure 5A**. For BP terms, the represented categories were immune system processes (GO: 0002376), regulation of immune system processes (GO: 0002682) and small molecule metabolic processes (GO: 0044281). For CC terms, the represented categories were extracellular region (GO: 0005576), extracellular region part (GO: 0044421) and extracellular organelle (GO: 0043230). For MF terms, the represented categories were oxidoreductase activity (GO: 0016491), hormone activity and carboxylic acid binding (GO: 0031406) (**Figure 5A**). A number of unigenes were also involved in binding, oxidoreductase activity and immune immunologic systems and anti-inflammation signaling, which suggested that these unigenes may play a role in EA protection and associated impacts. We selected the top 30 DEGs from each subgroups based on significance (*P*-value). Heatmaps of the top 30 DEGs were plotted using their log2-transformed FPKM values (Red represented upregulation; Blue represented downregulation in **Figure 5B**).

Many of the DEGs were found to play a role in redox, immunity, lipid metabolism and inflammation. DEGs in mice from groups D and E were further annotated by KEGG, which found that 15 pathways were significant (*P* < 0.05). **Figure 5C** showed KEGG enrichment analysis of DEGs. Compared with whole genome expression, the PPAR signaling pathway was found to be the most significantly enriched pathway.

### Validation of RNA-Seq Results with qRT-PCR

**Figure 6:**
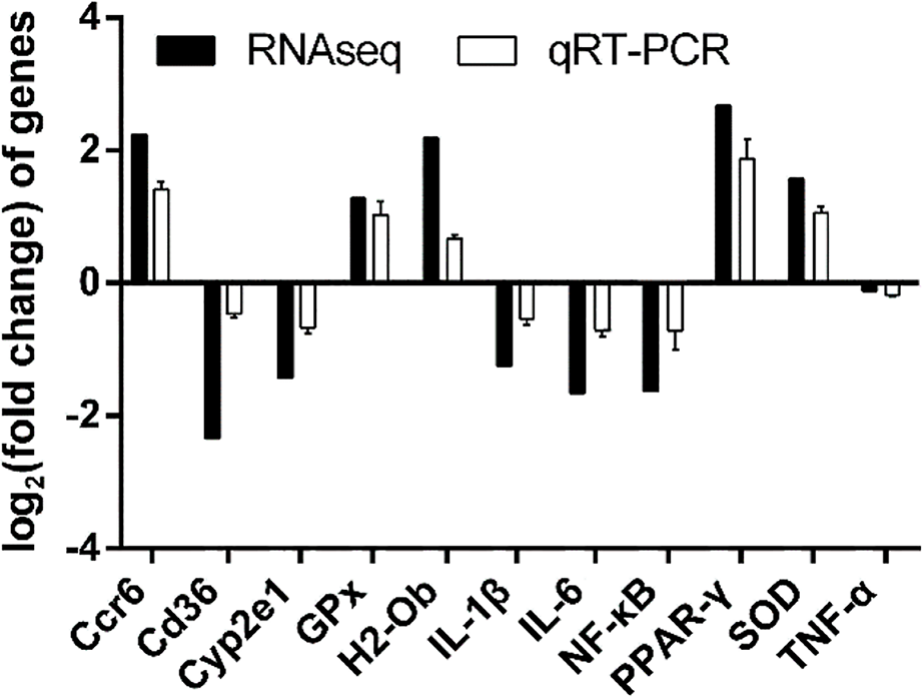
Comparison of DEGs and qRT-PCR confirmation.

The 3 genes (Cyp2e1, SOD and GPx) were identified to be associated with oxidative stress. The 5 genes (CD36, NF-κB, IL-1β, IL-6, and TNF-α) were identified to be associated with inflammation. The 2 genes (H2-Ob and Ccr6) were identified to be associated with immune functions. Fold-changes in qRT-PCR were compared with RNA-Seq expression profiles. The log2 (fold-change) values of the genes identified by RNA-seq and qRT-PCR were: Ccr6 (2.24 vs. 1.41), CD36 (–2.34 vs. –0.64), Cyp2e1 (–1.43 vs. –0.67), GPx (1.28 vs. 1.03), H2-Ob (2.18 vs. 0.67), IL-1β (–1.25 vs. –0.54), IL-6 (–1.66 vs. –0.72), NF-κB (–1.64 vs. –0.72) PPAR-γ (2.76 vs. 1.87), SOD (1.57 vs. 1.06) and TNF-α (–0.12 vs. –0.18). As shown in **Figure 6**, the qRT-PCR results were consistent with the high-throughput sequencing data, suggesting that the transcriptome sequencing data was reliable.

## Discussion

Diarrhea is a disease which can cause death in humans (Emerson and Savage, 2017) and animals (Wolff et al., 2017). Transcriptome analysis was used to study the mechanism of action of anti-diarrheal drugs (Men et al., 2018; Wu et al., 2018; Yao et al., 2018), while the mechanism of action of EA in the treatment of diarrhea remains unclear. In this study, we used EA to protect small intestine against castor oil-induced diarrhea in a mouse model. Deep transcriptome sequencing was used to analyze the transcriptomic profiles of ileum tissues in diarrhea model with or without EA treatment. We identified 273 DEGs, including 160 up-regulated genes and 113 down-regulated genes. DEGs were annotated by GO in mice from group E compared with group D. A total of 458 GO terms between groups D and E were significantly enriched. According to *P*-value, the top 30 DEGs were selected from each sub-category (BP, CC, and MF). There were 15 pathways annotated by KEGG in mice from group E compared with group D. PPAR-γ was the most significant signaling pathway. In the study, we used transcriptome to study the mechanism of EA protecting ileum and inhibiting diarrhea.

Many hazardous materials induced oxidative stress that caused inflammation or injury (Wang et al., 2019; Qu et al., 2019). In our study, Castor oil induced oxidative stress, decreased SOD and GPx levels, and increased MDA levels in group D compared with group C and E. Celik et al. (2013) reported that EA decreased the activity of Cyp2e1 and increased the activity of GPx (Celik et al., 2013). Jabri et al. (2016b) reported similar results (Jabri et al., 2016b). SOD and GPx activities were increased in EA-treated V79-4 cells (Han et al., 2006). Expression of PPAR-α was increased, whereas that of Cyp2e1 was reduced (Nakamuta et al., 2005; Aristatile et al., 2014). Yuce et al. (2007) found that the administration of EA to cisplatin‐treated rats decreased the MDA levels, and increased GPx and CAT in liver and heart tissue of rats (Yuce et al., 2007). In our experiment, EA increased expressions of PPAR-γ, SOD, GPx and decreased MDA level and Cyp2e1 expression, which suggested that EA decreased oxidative stress by PPAR signaling pathway. Cd36 is a membrane glycoprotein, which presented on some epithelia and contributed to inflammatory responses (Silverstein and Febbraio, 2009). Cd36 was a key modulator of proinflammatory and oxidative pathways. Silverstein and Febbraio (2009) found that Cd36-deficient mice exhibited levels of activated NF-κB and oxidative stress decreased in chronic kidney disease (CKD) (Silverstein and Febbraio, 2009). In our experiment, EA decreased Cd36, NF-κB, IL-1β, IL-6, and TNF-α, which suggested that EA can inhibit inflammation, protect the small intestine and treat diarrhea. Ccr6 was expressed on immature dendritic cells and B cells and memory T cells and involved in mucosal immune responses (Puleston et al., 2005), and a common marker for Th1/Th17 cells, which also preferentially expressed the nuclear receptor PPAR-γ (Bernier et al., 2013). The non-classical major histocompatibility complex (MHC) class II gene H2-Ob was enriched in antigen processing/presentation pathways (Stables et al., 2011). Additionally, H2-Ob is thought to be a switching gene in innate immunity (Khayer et al., 2017). In our experiment, EA increased Ccr6 and H2-Ob expression. Ramirez et al. (2017) reported that tea made from *Mangifera indica* L. leaves of the Uba variety upregulated PPAR-γ, exerting antioxidant and anti-inflammatory effects (Ramirez et al., 2017). PPAR regulated the genes of Ccr6, Cd36, Cyp2e1, and H2-Ob. EA activated PPAR signaling pathway and treated diarrhea, by Inhibiting oxidative stress, inhibiting inflammation and improving immunity. Interestingly, GW9662, a PPAR antagonist, inhibited the effect of EA on diarrhea and inflammation in group V, which suggested that EA treatmented of diarrhea through PPAR signaling pathway. Yang et al. (2014) reported that some PPAR-γ ligands, such as emodin and protocatechuic acid, were used to attenuate inflammation by activating PPAR-γ, which was activated by the MAPK/NF-κB pathway (Yang et al., 2014). Furthermore, activated NF-κB mediated the expression of a number of rapid response genes, such as iNOS and COX-2, involved in the inflammatory response to injury (Shi et al., 2019; Hu et al., 2019);. The expressions of COX-2 and iNOS can increased the expression of IL-1β, IL-6, and TNF-α (Horie et al., 2009). Consistent with this notion, our result of RNA sequencing showed that EA increased the expressions of PPAR-γ and decreased the expressions of IL-1β, IL-6, NF-κB, and TNF-α, which were proved by qRT-PCR.

In conclusion, transcriptome analysis of the ileum in a mouse diarrhea model successfully identified a large number of DEGs. These genes showed that EA mainly treated diarrhea by activating the PPAR signaling pathway.

## Data Availability Statement

The datasets generated for this study can be found in NCBI GEO accession GSE142063.

## Ethics Statement

All experimental processes about animals complied with EU Directive (2010/63/EU) and were approved by the Ethics Committee of Northeast Agricultural University of China (Protocol number: SRM-06).

## Author Contributions

JC did the experiment and wrote the manuscript. HY and ZS designed and funded this experiment.

## Funding

This work was supported by the National Natural Science Foundation of China (Grant No. 31572559) and (Grant No. 31802227); Postdoctoral scientific research developmental fund of Heilongjiang Province in 2018 (LBH-Q18020).

## Conflict of Interest

The authors declare that the research was conducted in the absence of any commercial or financial relationships that could be construed as a potential conflict of interest.
